# Aberrant expression of multiple T cell markers on diffuse large B cell lymphoma: a case report

**DOI:** 10.1186/s43046-021-00071-7

**Published:** 2021-06-15

**Authors:** Anurag Mehta, Prerna Chadha, Poojan Agarwal, Sunil Pasricha

**Affiliations:** 1 Department of Laboratory and Transfusion Services, Rajiv Gandhi Cancer Institute & Research Centre, Sector 5, Rohini, Delhi, 110085 India; 2 Department of Research, Rajiv Gandhi Cancer Institute & Research Centre, Delhi, India

**Keywords:** Diffuse large B cell lymphoma, Aberrant antigen expression, CD 3

## Abstract

**Background:**

Aberrant T cell antigen expression has been well documented in diffuse large B cell lymphomas. However, co-expression of multiple T cell antigens including CD3, which has been considered a specific marker for T cells is extremely rare. Awareness about such aberrant expression is important so as not to misdiagnose or wrongly classify a lymphoma. The aim of this article is to report such a case.

**Case presentation:**

A 68-year-old postmenopausal lady, diabetic and hypertensive, presented with an axillary lump of one week’s duration. There was no other relevant medical history. Ultrasonography revealed multiple hypoechoic cystic lesions varying in size from 3.9 to 4.2 cm^3^. Aspiration was suggestive of an infective pathology. Excision biopsy of the mass was diagnosed as diffuse large B cell lymphoma with aberrant T cell antigen expression. She received 4 cycles of chemotherapy after which she was lost to follow-up.

**Conclusion:**

The case presented as a diagnostic dilemma for the pathologist. The predicament lies in classifying it as a B cell lymphoma with an aberrant expression of T cell markers versus a T cell lymphoma with an aberrant B cell marker expression which has a significant implication on the treatment offered. This can be solved by looking at the expression of the B cell specific transcription factors. The key to diagnosis lies in the knowledge of their existence and the application of a panel of markers.

**Supplementary Information:**

The online version contains supplementary material available at 10.1186/s43046-021-00071-7.

## Background

Diffuse large B cell lymphomas (DLBCLs) are the most common of all non-Hodgkin lymphomas (NHLs). They are defined as neoplasms of large lymphoid cells with a nuclear size equal to or more than that of a macrophage nucleus, or nuclear size more than twice the size of a normal lymphocyte [1]. Most cases of DLBCLs show immunohistochemical expression of B cell antigens such as CD 19, CD 20, and CD 79a. They may be classified into germinal center or non-germinal center types according to Hans algorithm based on the expression of CD 10, Bcl 6, and IRF4/MUM1. Variable expression of FOXP1, and less commonly CD 5 has also been observed. However, expression of T cell-associated antigens especially CD 3, which has largely been considered as a marker specific for T cell lymphomas, is exceedingly rare in B cell NHLs [2]. One such case of DLBCL, expressing multiple T cell-associated antigens, including CD 4, CD 2, CD 7, CD 43, and CD 3, is described here.

## Case presentation

A 68-year-old postmenopausal lady, diabetic and hypertensive, presented with a right axillary lump the size of a small tennis ball of 1 week’s duration. No other significant medical history was offered by the patient. Local examination revealed a well circumscribed, tender, and mobile lump which was not tethered to the overlying skin or underlying soft tissue. The lump was rubbery in consistency and measured approximately 5 × 5 × 4 cm. The general physical and systemic examinations were non-contributory. There was no evidence of any organomegaly.

The lab workup at our hospital showed a hemoglobin of 8.9g/dl and lactate dehydrogenase (LDH) level within normal range (65 U/L). Ultrasonography of the axilla revealed multiple hypoechoic cystic areas in the right axilla varying in size from 3.9-4.2 cm^3^. Aspirate performed was suppurative and suggestive of an infective pathology. An excision biopsy of the mass was performed and the pathological findings are as below.

### Pathological findings

**Fig. 1:**
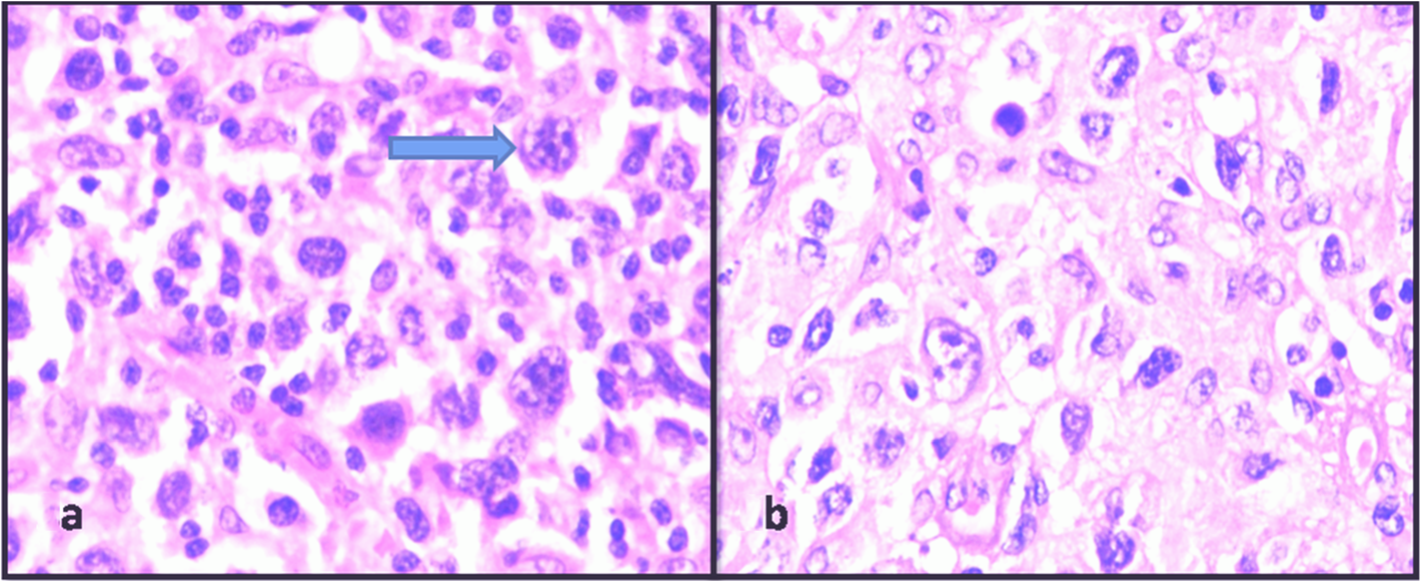
Histology: **a**, **b** H&E stained section showing a polymorphous population of cells with numerous multilobated cells (arrow)

Sections from the axillary lump sent as “breast tissue” showed it to be an effaced lymph node. The lymphoid tissue was composed of predominantly large cells in sheets, constituting a tumor. The individual tumor cells had moderate amount of eosinophilic cytoplasm with ovoid vesicular nuclei having clumped chromatin and prominent nucleoli. Multinucleate cells, binucleate cells, and bizarre forms with polylobated nuclei were noted (Fig. 1a, b). These cells were admixed with a dense mixed inflammatory cell infiltrate composed of lymphocytes, plasma cells, and histiocytes. Large areas of necrosis with karyorrhectic debris were seen. Mitosis was brisk and atypical forms were noted. Apoptotic bodies were evident. The periphery of the tissue showed residual lymphoid follicles. No native breast parenchyma was identified.

**Fig. 2:**
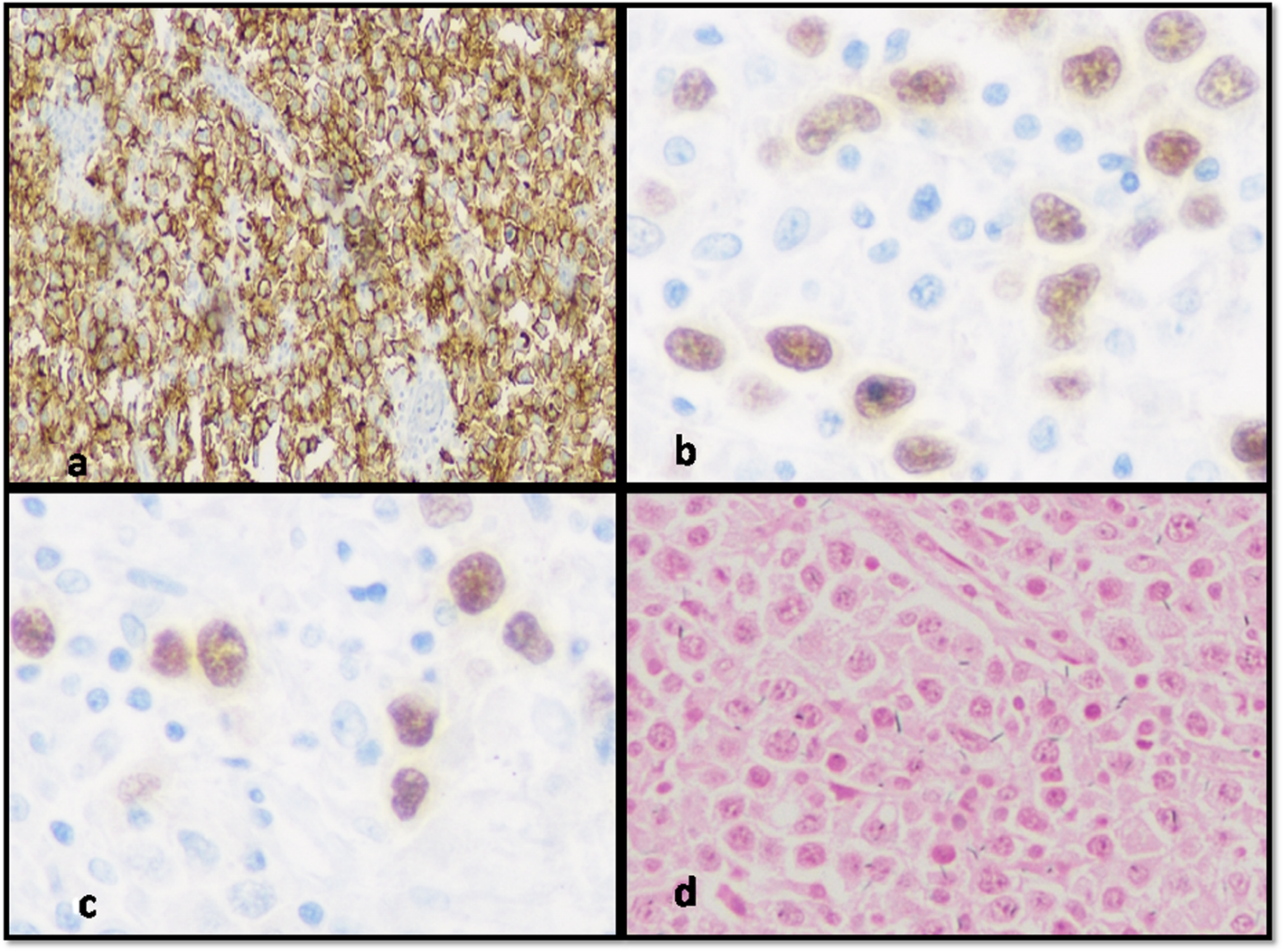
Immunohistochemistry for B cell markers. **a** The tumor cells show strong and diffuse membranous staining for CD 20. **b**, **c** Nuclear positivity for PAX5 and OCT2 respectively in the polylobated cells. **d** EBER by ISH is negative

**Fig. 3:**
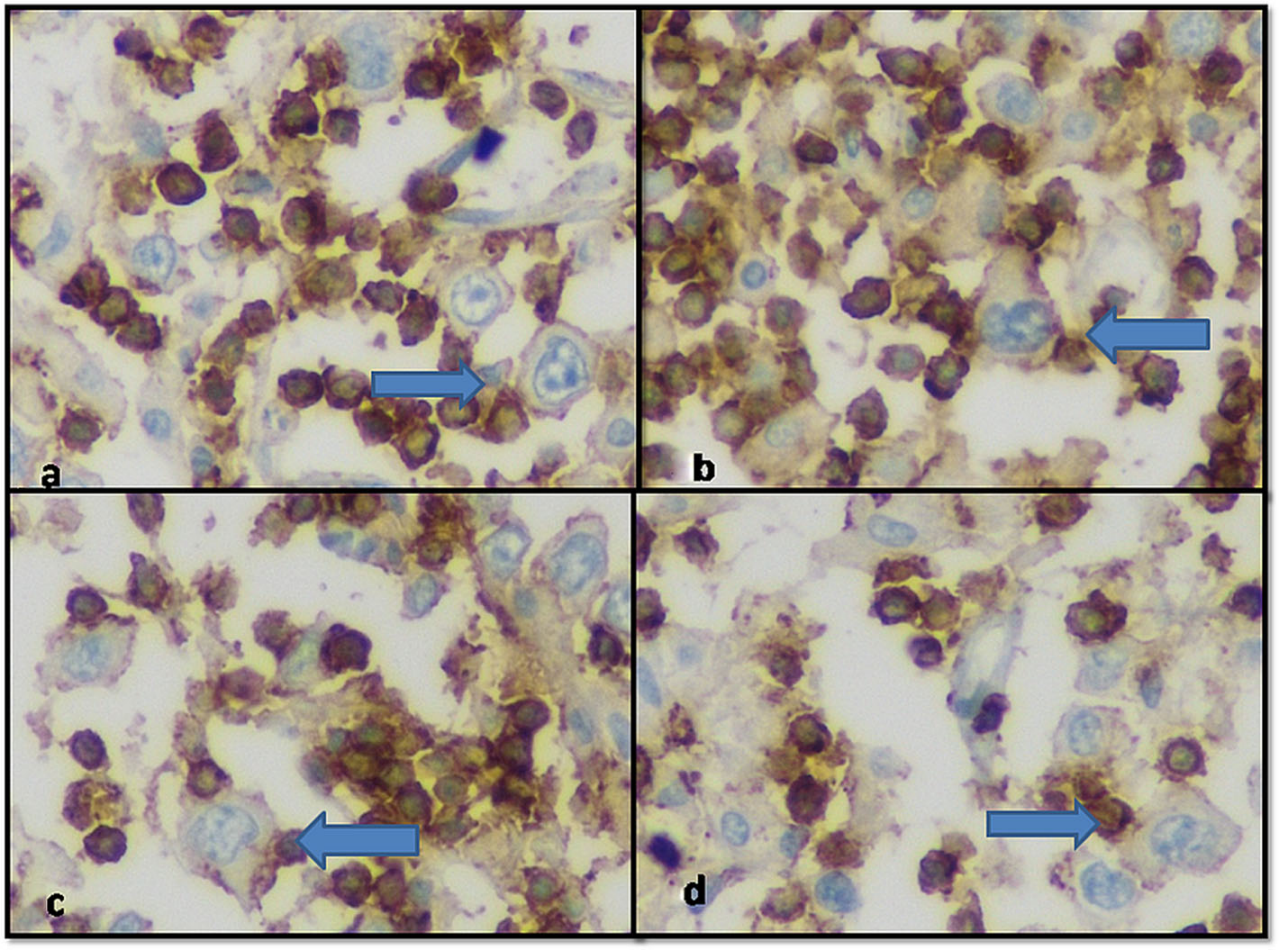
Aberrant expression of T cell markers on immunohistochemistry. **a**, **b**, **c**, **d** Weak membranous staining for CD4, CD2, CD7, and CD 3 respectively in the large tumor cells (arrow)

On immunohistochemistry (IHC), the tumor cells were immunopositive for LCA, CD 20 (Fig. 2a), PAX 5 (diffuse and strong), Oct 2 (Fig. 2b, c), BOB 1 (weak), and CD 30 (focal) while being immunonegative for CK, S100, CD 68, CD8, CD 5, and CD 15. The tumor cells also showed immonopositivity for CD 43, CD 4 (Fig. 3a), CD 2 (Fig. 3b), CD 7 (Fig. 3c), and CD 3 (aberrant, weak) (Fig. 3d). CD 23 highlighted the follicular dendritic cell (FDC) meshwork of residual lymphoid follicles. Ki 67 labeling index was 70%. Epstein-Barr encoding region (EBER) by in situ hybridization (ISH) was negative in the neoplastic population (Fig. 2d).

A final diagnosis of DLBCL with aberrant expression of T cell-associated antigens (ABTCE), namely, CD 4, CD 2, CD 43, CD 7, and CD 3, was given. She received 4 cycles of rituximab, cyclophosphamide, doxorubicin, vincristine, and prednisolone (R-CHOP)-based chemotherapy and was evaluated as having partial response to therapy after which she was lost to follow-up.

## Discussion

ABTCE in B cell NHLs is not a common occurrence but has been reported in certain studies [3]. It has most commonly been seen in association with chronic lymphocytic leukemia and mantle cell lymphoma [2]. According to researchers, the overall frequency of expression of T cell antigens in DLBCLs was only 7.3% with CD 2 and CD 7 being the ones most commonly expressed [4]. ABTCE has also been reported for CD 7, CD 5, CD 2, CD 4, and/or CD 8 [5–8]. Moreover, two or more T cell antigens have been reported together, as described in Table 1.

**Table 1:** Aberrant T cell marker expression in B cell lymphomas

Researchers	Aberrant T cell marker expressed				
	CD2	CD4	CD5	CD7	CD8
Yoshida et al. [4]	+	−	−	+	−
Inaba et al. [7]	+	−	−	+	−
Schmidt et al. [8]	+	−	−	+	−
Sangle et al. [9]	+	−	−	+	−
Hussaini et al. [2]	+	−	+	−	−
Kaleem et al. [6]	+	−	−	−	+
Sangle et al. [10]	−	+	+	−	+

However, literature pertaining to the expression of multiple T cell antigens including CD 3 is extremely rare. Both Kaleem et al. [6] and Suzuki et al. [4] found no case of DLBCL expressing CD 3 in their respective studies on B cell NHLs. To the best of our knowledge, this is the first case of a DLBCL with the aberrant expression of multiple T cell-associated antigens, namely, CD 2, CD 4, CD 7, CD43, and CD 3.

ABTCE has been demonstrated most often on flow cytometry [4, 6]. Only a few studies have used immunopositivity on IHC to prove the ABTCE in DLBCLs [5, 11, 12]. This report also uses IHC for the demonstration of ABTCE.

DLBCLs showing ABTCE are usually seen in association with chronic inflammation [4]; wherein, EBV is considered to contribute to this phenomenon. However, our case was negative for EBER as demonstrated on ISH, indicating that there might be other factors contributing to ABTCE. In literature, CD2 or CD7 expression is reported to be associated with extranodal involvement in B-NHL at diagnosis [7]. However, this was not seen in our case.

The histopathology of DLBCL with ABTCE is usually not different from that of other DLBCLs, and shows sheets of large atypical cells with ovoid vesicular nuclei and prominent nucleoli. However, our case was characterized by an abundance of reactive lymphoid tissue and endothelial proliferation as is usual in peripheral T cell lymphomas in addition to the diffuse sheets of large atypical cells. This epiphenomenon may be attributable to the aberrant T cell phenotype inviting reactive cells.

Based on the immunohistochemical expression of CD 20 and PAX5 (diffuse and strong) along with the expression of B cell specific transcription factors such as OCT2 and BOB1, a diagnosis of DLBCL was given despite the immunopositivity for multiple T cell antigens including CD3. The case being reported is a non-Hodgkin lymphoma with no peripheralization. Though mixed phenotypes have often been reported in literature in acute leukemia, no such report exists of a mixed leukemia with large cell morphology [13]. Hence, the diagnosis of mixed phenotypic leukemia was not entertained despite expression of CD 3 (clone SP 7 against epsilon chain). Expression of multiple B cell markers with an intact B cell transcription apparatus as demonstrated by the simultaneous expression of OCT2, BOB1, PAX5, and BCL6 amply defines the true B cell lineage [14]. However, α/β T cell receptor rearrangement would contribute significantly in negating the possibility of mixed phenotype lymphoma conclusively, which was not performed in this case.

There are a few hypotheses to explain such ABTCE in B cell lymphomas. Some researchers believe that the deregulated control of gene expression in malignant B cells, leads to the activation of some silent or repressed genes of T cell differentiation [6], while other researchers have found an association with EBV whose latent membrane protein-1 was postulated to have a modulatory effect on the expression of T cell molecules [4].

Clinically, the results of patients with ABTCE in B cell lymphomas are ambiguous. Some studies show that they have a more aggressive disease course [7] while others do not show any relation of ABTCE to survival [4]. Our patient was lost to follow-up after achieving a partial response to therapy.

To conclude, the importance of recognizing ABTCE in B cell NHLs lies in the fact that these cases might prove to be a dilemma for the pathologist as to classify them as a composite lymphoma or a lymphoma with an aberrant marker expression. Another predicament lies in classifying them as B cell lymphomas with an ABTCE versus a T cell lymphoma with an aberrant B cell marker expression. This can be solved by looking at the expression of the B cell specific transcription factors such as OCT 2 and BOB 1, immunopositivity for which leads to the preferred diagnosis of a B cell NHL with ABTCE, as was seen in our case. The key to recognizing such cases, lies in the knowledge of their existence, and the practice of applying a panel of markers. This case represents one such example and contributes to the scarce literature regarding the aberrant expression of multiple T cell antigens in DLBCLs, especially CD 3. This study is limited by a lack of follow-up and performing gene expression or rearrangement studies to conclusively negate the possibility of mixed phenotype acute leukemia/lymphoma (B/T). However, the objective of this study is to highlight the expression of multiple routinely employed antibodies of T lineage being concurrently seen in DLBCL. We also wanted to share the fact that several T cell antigens can simultaneously be expressed aberrantly along with the primary B cell immunophenotype. Demonstrating an intact B cell transcription apparatus with CD20 expression may be enough to call it as DLBCL and treat the patient with chemoimmunotherapy providing the benefit of rituximab to achieve the best outcome.

## Supplementary Information

**Additional file 1..**

## Data Availability

Not applicable.

## Abbreviations

DLBCL: Diffuse large B cell lymphoma
NHL: Non-Hodgkin lymphoma
LDH: Lactate dehydrogenase
H&E: Hematoxylin and eosin
IHC: Immunohistochemistry
FDC: Follicular dendritic cell
EBER: Epstein-Barr encoding region
ISH: In situ hybridization
ABTCE: Aberrant T cell antigen expression
RCHOP: Rituximab, cyclophosphamide, doxorubicin, vincristine, and prednisolone
PET CT: Positron emission tomography-computed tomography
