# Supplementary figures and images for: Aberrant expression of multiple T cell markers on diffuse large B cell lymphoma: a case report
Source: J Egypt Natl Canc Inst. 2021 Jun 15;33:14. doi: 10.1186/s43046-021-00071-7 (PMC13316921; doi:10.1186/s43046-021-00071-7)

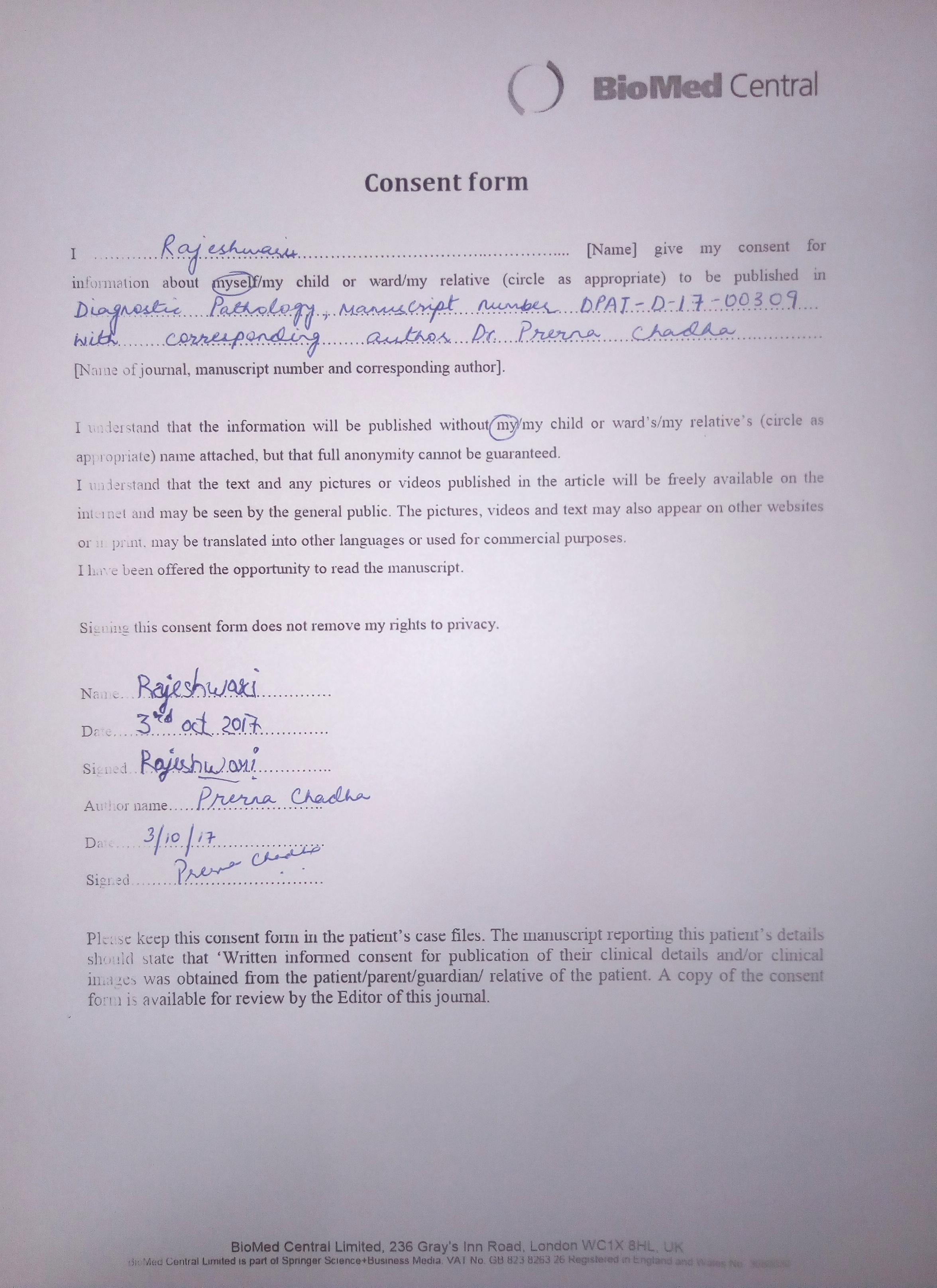

Supplement: Supplementary file 1 — Additional file 1.. [file 43046_2021_71_MOESM1_ESM.zip › consent form dlbl.jpg]

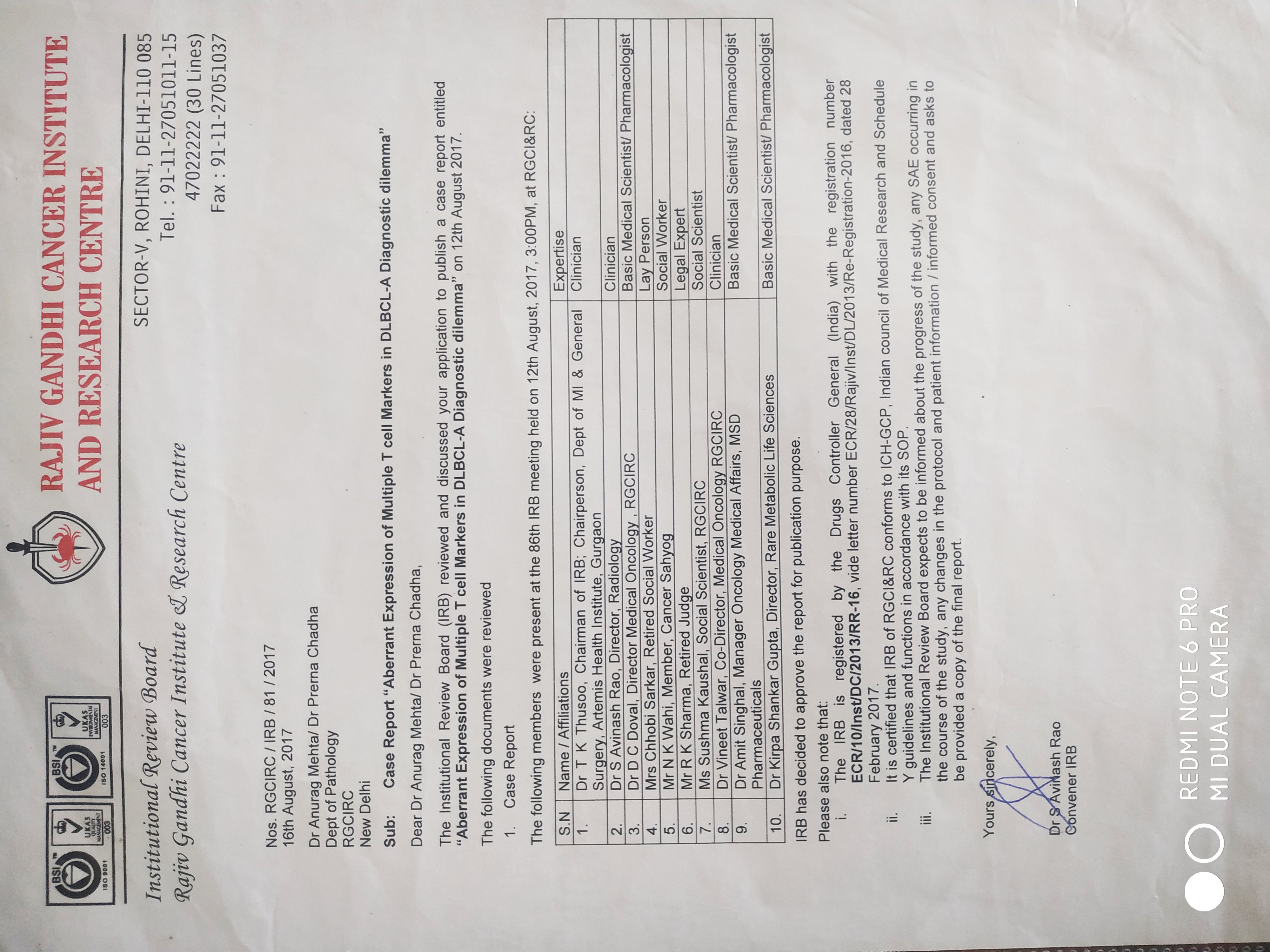

Supplement: Supplementary file 1 — Additional file 1.. [file 43046_2021_71_MOESM1_ESM.zip › irb dlbl.jpg]
